# The Emerging Role of Vitamin C in the Prevention and Treatment of COVID-19

**DOI:** 10.3390/nu12113286

**Published:** 2020-10-27

**Authors:** Anitra C. Carr, Sam Rowe

**Affiliations:** 1 Nutrition in Medicine Research Group, Department of Pathology & Biomedical Science, University of Otago, Christchurch 8011, New Zealand; 2 Intensive Care Department, Newham University Hospital, Barts NHS Trust, London E13 8SL, UK; sam.rowe2@nhs.net; 3 Clinical Sciences, Liverpool School of Tropical Medicine, Liverpool L3 5QA, UK

**Keywords:** vitamin C, ascorbate, ascorbic acid, COVID-19, pneumonia, sepsis, acute respiratory distress syndrome, randomized controlled trials, low-middle-income

## Abstract

Investigation into the role of vitamin C in the prevention and treatment of pneumonia and sepsis has been underway for many decades. This research has laid a strong foundation for translation of these findings into patients with severe coronavirus disease (COVID-19). Research has indicated that patients with pneumonia and sepsis have low vitamin C status and elevated oxidative stress. Administration of vitamin C to patients with pneumonia can decrease the severity and duration of the disease. Critically ill patients with sepsis require intravenous administration of gram amounts of the vitamin to normalize plasma levels, an intervention that some studies suggest reduces mortality. The vitamin has pleiotropic physiological functions, many of which are relevant to COVID-19. These include its antioxidant, anti-inflammatory, antithrombotic and immuno-modulatory functions. Preliminary observational studies indicate low vitamin C status in critically ill patients with COVID-19. There are currently a number of randomized controlled trials (RCTs) registered globally that are assessing intravenous vitamin C monotherapy in patients with COVID-19. Since hypovitaminosis C and deficiency are common in low–middle-income settings, and many of the risk factors for vitamin C deficiency overlap with COVID-19 risk factors, it is possible that trials carried out in populations with chronic hypovitaminosis C may show greater efficacy. This is particularly relevant for the global research effort since COVID-19 is disproportionately affecting low–middle-income countries and low-income groups globally. One small trial from China has finished early and the findings are currently under peer review. There was significantly decreased mortality in the more severely ill patients who received vitamin C intervention. The upcoming findings from the larger RCTs currently underway will provide more definitive evidence. Optimization of the intervention protocols in future trials, e.g., earlier and sustained administration, is warranted to potentially improve its efficacy. Due to the excellent safety profile, low cost, and potential for rapid upscaling of production, administration of vitamin C to patients with hypovitaminosis C and severe respiratory infections, e.g., COVID-19, appears warranted.

For a quarter of a century, it has been known that critically ill patients, including those with sepsis and multiple organ failure, have very low vitamin C status [1,2,3]. It has also been demonstrated that these critically ill patients have higher requirements for vitamin C, with gram doses required to normalize their blood levels [4,5], 20–30 times more than is required for the general population. Despite these findings, critically ill patients with sepsis continue to be administered milligram amounts of vitamin C, which is insufficient to replete their vitamin C status [6]. In 2014, Dr Fowler and colleagues published the findings of a small clinical trial which indicated that intravenous administration of gram amounts of vitamin C to patients with sepsis could improve organ failure scores and decrease markers of inflammation (C-reactive protein) and tissue damage (thrombomodulin) [7]. In a larger randomized controlled trial (RCT) of septic patients with acute respiratory distress syndrome (ARDS), the CITRIS-ALI trial, intravenous administration of 200 mg/kg/d of vitamin C for 4 days resulted in a 28 day mortality of 30% relative to 46% in the placebo group (*p* = 0.03) and a hazard ratio (HR) of 0.55 (95% CI, 0.33–0.90, *p* = 0.01) [8]. The number of intensive care unit (ICU)- and hospital-free days was also significantly higher in the vitamin C group.

Administration of vitamin C late in the disease process, e.g., when ARDS has developed, likely attenuates its effectiveness. Earlier clinical trials have indicated that administration of vitamin C to patients with pneumonia can decrease the severity of the respiratory symptoms, particularly of the most severely ill patients, and the duration of hospital stay [9,10]. Thus, administration of vitamin C earlier in the respiratory infection process may prevent its progression to sepsis [11]. Survival data from the CITRIS-ALI trial has indicated that the effect of vitamin C on survival is most apparent during the 4 day infusion period [12]. Furthermore, pharmacokinetic research has indicated that upon cessation of vitamin C infusion, the vitamin C status of some patients returns to their low pre-infusion levels [5]. These findings call to sustained administration of the vitamin in the ICU. This will likely also improve the long-term outcomes of the patients, particularly if they continue to take the vitamin orally following discharge from ICU, due to its important roles in immunological function and in multiple organ systems [11]. 

Earlier this year, Dr Fowler and colleagues published a review on the emerging role of vitamin C as a treatment for sepsis [13]. In this review, they summarised the current state of knowledge around its pleiotropic physiological functions in sepsis. These include its roles as an antioxidant, a cofactor for the synthesis of vasopressors (norepinephrine and vasopressin), and roles in leukocyte and platelet functions, and endothelial and epithelial cell integrity. In the face of the current severe acute respiratory syndrome coronavirus (SARS-CoV-2) pandemic, this review has been very timely, with sepsis being a significant complication of severe coronavirus disease (COVID-19) [14]. 

Many of the functions of vitamin C appear relevant to COVID-19-related sepsis and ARDS. For example, recent research has uncovered a connection between SARS-CoV-2 infection and depleted levels of the antiviral cytokine interferon [15], and a negative association between interferon levels and disease severity [16,17]. Of note, vitamin C has been shown to augment interferon levels in animal models of viral infection [18,19]. Another characteristic of severe COVID-19 is elevated inflammatory markers and this can present as a ‘cytokine storm’ in some cases [14]. Vitamin C has anti-inflammatory and antioxidant activities which can potentially counteract this phenomenon [13]. Preliminary evidence from a small COVID-19 trial indicates that administration of intravenous vitamin C can significantly decrease IL-6 levels by day 7 of infusion [20]. 

Other common complications of COVID-19 are coagulopathy and microthrombi formation [14], which is likely a major component of COVID-19 lung pathology [21]. Early injection of vitamin C has been shown to prevent microthrombi formation and capillary plugging [22], and a case series has shown decreased D-dimer levels in COVID-19 patients who were administered intravenous vitamin C [23]. Neutrophil extracellular traps (NETs) have been implicated in COVID-19-related thrombotic complications [24,25]. Previous research has indicated that vitamin C administration can attenuate NETs in sepsis models [26], and post-hoc analysis of the CITRIS-ALI trial indicated decreased circulating cell-free DNA 48 h after administration of intravenous vitamin C [27]. Neutrophil-derived oxidative stress is believed to induce tissue damage in COVID-19 [28,29]. Patients with pneumonia and sepsis have significantly elevated oxidative stress markers relative to other critically ill patients [30,31], and early studies indicated that administration of vitamin C and other antioxidants to patients with septic shock and ARDS stabilized oxidative stress markers and improved cardiovascular parameters and survival [32,33].

In March, the World Health Organization published a coordinated global research roadmap for the 2019 novel coronavirus in which they identified a number of scientific knowledge gaps around determination of interventions that improve the clinical outcome of COVID-19-infected patients, including optimal selection of strategies for supportive care of seriously ill patients [34]. Of note, vitamin C was highlighted as an adjunctive intervention with biologic plausibility. Meta-analyses of relevant clinical trials have indicated that administration of vitamin C to critically ill patients can decrease the duration of mechanical ventilation and length of stay in ICU [35,36]. This is pertinent given global shortages of ICU capacity and may be particularly important for resource-limited settings such as those found in low–middle-income countries (LMICs) [37]. Of note, vitamin C production could be rapidly up-scaled globally, unlike many of the novel pharmacological treatments, some of which, e.g., remdesivir, have global shortages.

It is noteworthy that a majority of the top 10 countries with the highest COVID-19 case-loads are LMICs. In a recent review we highlighted the high prevalence of hypovitaminosis C and deficiency in LMICs (Figure 1) [38]. Furthermore, many of the risk factors for COVID-19 overlap with risk factors for vitamin C deficiency, such as poverty [39,40]. People who already have hypovitaminosis C are particularly susceptible to developing outright deficiency, and ergo more likely to respond to vitamin C administration. Therefore, the baseline vitamin C status of people with COVID-19 will likely affect their outcomes and their response to intervention [41,42]. 

As yet, there have been few publications reporting on the vitamin C status of patients with COVID-19. An observational study in the USA has indicated a mean vitamin C status of 22 ± 18 µmol/L in a cohort of 21 critically ill patients with COVID-19 [43], which is comparable to other studies of critically ill patients with sepsis [11]. An earlier case series from Spain indicated the absence of any detectable vitamin C in 17 of a cohort of 18 COVID-19 patients with ARDS [44]. However, the veracity of the methodology used to assess the vitamin C status of the patients was not clear, so the values could be artifactually low [45]. 

There are a number of clinical trials registered globally assessing intravenous vitamin C monotherapy in COVID-19 patients (Table 1). The first trial off the ground was in Hubei, China (NCT04264533) [46]. In this trial the investigators planned to treat 140 patients with a placebo control or intravenous vitamin C at a relatively high dose of 24 g/day for 7 days, and assess requirements for mechanical ventilation and vasopressor drugs, organ failure scores, ICU length of stay and mortality. A preprint of the findings of 54 patients from this trial is currently under peer review [15]. Although the investigators reported no differences between the treatment and placebo groups for the above outcomes, when a subgroup of the most severely ill patients (SOFA scores ≥ 3) was assessed, a significant decrease in ICU and hospital mortality was observed in the vitamin C group—18% vs. 50% in the placebo group (HR 0.2, 95% CI 0.1–0.9, *p* = 0.03). Unfortunately, the baseline vitamin C status of the patients was not reported.

The USA currently has the highest number of COVID-19 cases globally, however despite this, there are only a couple of small intravenous vitamin C monotherapy and COVID-19 trials registered in the USA (NCT04344184 and NCT04363216). A trial at the Cleveland Clinic is assessing oral vitamin C with and without zinc (NCT04342728). However, due to the pharmacokinetics of enteral and parenteral vitamin C differing dramatically, and the higher requirement for vitamin C during respiratory infections, oral vitamin C may not be as efficacious as intravenous vitamin C [42]. As such, future meta-analyses of vitamin C and COVID-19 clinical trials should include subgroup analyses of studies comprising intravenous vs. oral vitamin C. Furthermore, subgroup analysis of low vitamin C populations could also be carried out as RCT findings will likely vary depending on the country and hence the baseline vitamin C status of the population in which the study was carried out [47]. In populations with a high prevalence of chronic hypovitaminosis C, vitamin C intervention may show greater efficacy.

Overall, vitamin C exhibits plausible mechanisms of action that are of relevance to severe respiratory infection, including antioxidant, anti-inflammatory, antithrombotic, and immuno-modulatory functions. Based on the findings from clinical trials of patients with pneumonia and sepsis, and preliminary observational and interventional studies of COVID-19 patients, it is likely that vitamin C administration will improve outcomes in COVID-19. The upcoming findings from the larger RCTs currently underway (e.g., LOVIT-COVID), including introduction of intravenous vitamin C arms to large adaptive trials (e.g., REMAP-CAP; NCT02735707), will provide more definitive evidence. Some of these trials (e.g., LOVIT-COVID) are also examining the longer-term quality of life effects of short-term vitamin C administration. Optimization of the intervention protocols in future trials, e.g., earlier and sustained administration, is warranted to potentially improve its efficacy. Due to the excellent safety profile, low cost, and potential for rapid upscaling of production, administration of vitamin C to patients with hypovitaminosis C and severe respiratory infections, e.g., COVID-19, appears warranted.

## Figures and Tables

**Figure 1:**
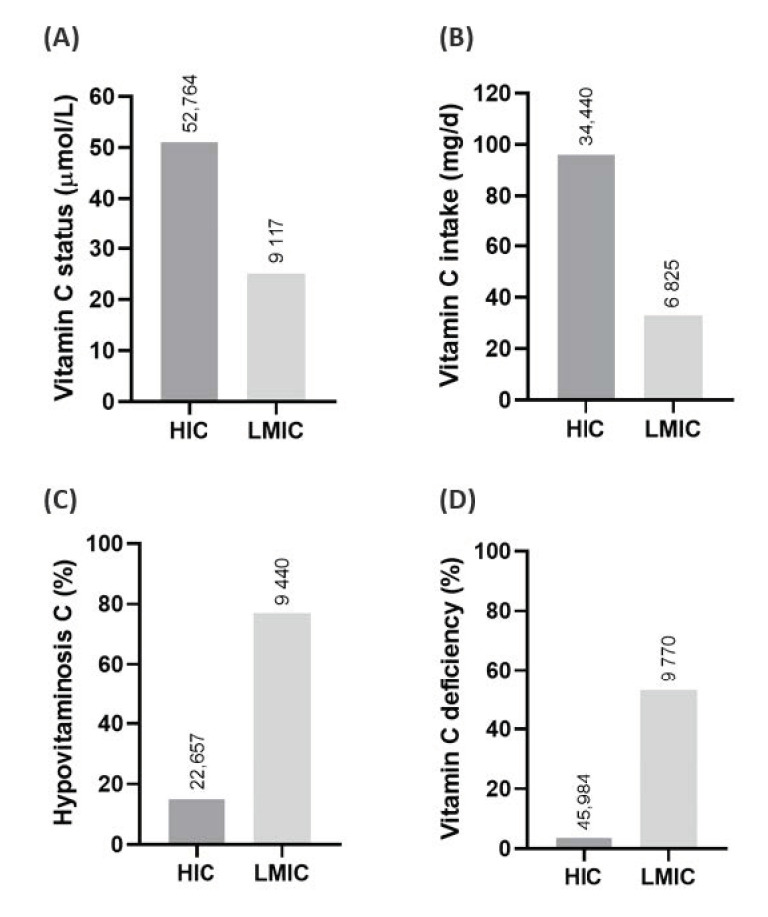
Summary of global vitamin C status (**A**) and intake (**B**) and prevalence of hypovitaminosis (**C**) and vitamin C deficiency (**D**). HIC—high-income countries; LMICs—low-middle-income countries. Hypovitaminosis C, <23 µmol/L; vitamin C deficiency, <11 µmol/L. Numbers above bars indicate the total number of individuals assessed. Data from [38].

**Table 1:** Summary of registered intravenous vitamin C (IVC) monotherapy and COVID-19 trials globally.

Country Study ID	Title	Participants	Intervention	Primary Outcome(s)
Canada NCT04401150	Lessening Organ Dysfunction with VITamin C—COVID-19 (LOVIT-COVID)	800 hospitalized patients with COVID-19	50 mg/kg/6 h IVC for 96 h vs. placebo	Death or persistent organ dysfunction
Italy NCT04323514	Use of Ascorbic Acid in Patients With COVID-19	500 patients with COVID-19 pneumonia	10 g/d IVC for 72 h uncontrolled	In-hospital mortality
USA NCT04344184	Early Infusion of Vitamin C for Treatment of Novel COVID-19 Acute Lung Injury (EVICT-CORONA-ALI)	200 patients with COVID-19 acute lung injury	100 mg/kg/8 h IVC for 96 h vs. placebo	Number of ventilator-free days
USA NCT04363216	Pharmacologic Ascorbic Acid as an Activator of Lymphocyte Signaling for COVID-19 Treatment	66 patients with COVID-19	0.3–0.9 g/kg/d IVC for 6 days vs. control	Clinical Improvement
China NCT04264533	Vitamin C Infusion for the Treatment of Severe 2019-nCoV Infected Pneumonia	140 patients with COVID-19 pneumonia	12 g/12 h IVC for 7 days vs. placebo	Ventilator-free days
China ChiCTR-2000032400	The efficacy and safety of high dose IVC in the treatment of novel coronavirus pneumonia (COVID-19)	120 patients with COVID-19 pneumonia	100 mg/kg/d IVC for up to 7 days vs. placebo	CRP, ESR, existence of SIRS
Iran IRCT2020-0411047025N1	Evaluation of effectiveness of IVC in Patients with COVID-19 Referred to Imam Khomeini Hospital	110 patients with COVID-19	1.5 g/6 h IVC for up to 5 days vs. control	Improvement of SPO_2_
Iran IRCT2019-0917044805N2	Effects of High-dose Vitamin C on Treatment, Clinical Symptoms and Laboratory Signs of Iranian COVID-19 Patients	60 patients with COVID-19	12 g/d IVC for 4 days vs. placebo	Time to clinical improvement
Iran IRCT2020-0516047468N1	Interventional study of IVC in definitive patients with COVID-19 and its effect on changes in lung CT scan and clinical and laboratory symptoms of patients	50 patients with COVID-19	2 g/6 h IVC for 5 days vs. control	The amount of lung involvement in a CT scan
